# HSPB1 as an RNA-binding protein mediates the pathological process of osteoarthritis

**DOI:** 10.1186/s13018-024-04580-8

**Published:** 2024-03-01

**Authors:** Qiang Fu, Yi Li, Chunhua Shi

**Affiliations:** Department of Rheumatology and Immunology, Jiangxi Provincial People’s Hospital, The First Affiliated Hospital of Nanchang Medical College, Nanchang, 330006 Jiangxi China

**Keywords:** Osteoarthritis, iRIP-seq, RNA-binding, EGFR, ARE

## Abstract

Heat-shock protein beta1 (HSPB1) is a member of the small HSP family, downregulated in osteoarthritis (OA) chondrocytes and demonstrated the capacity to serve as an RNA-binding protein (RBP). This work aimed to explore the profile of HSPB1 bound RNA and reveal the potential regulation mechanism of HSPB1 in OA. In this work, we captured an unbiased HSPB1-RNA interaction map in Hela cells using the iRIP-seq. The results demonstrated that HSPB1 interacted with plentiful of mRNAs and genomic location toward the CDS region. Functional enrichment of HSPB1-related peaks showed the involvement in gene expression, translation initiation, cellular protein metabolic process, and nonsense-mediated decay. HOMER software analysis showed that HSPB1 bound peaks were over-represented in GAGGAG sequences. In addition, ABLIRC and CIMS algorithm indicated that HSPB1 bound to AU-rich motifs and the proportion of AU-rich peaks in 3′ UTR were slightly higher than that in other regions. Moreover, HSPB1-binding targets analysis revealed several gens were associated with OA including EGFR, PLEC, COL5A1, and ROR2. The association of OA-related mRNAs to HSPB1 was additionally confirmed in OA tissues by the quantitative RIP-PCR experiments. Further experiment demonstrated the downregulation of HSPB1 in OA tissues. In conclusion, our current study confirmed HSPB1 as an RNA-binding protein and revealed its potential function in the pathological process of OA, providing a reliable insight to further investigate the molecular regulation mechanism of HSPB1 in OA.

**Supplementary Information:**

The online version contains supplementary material available at 10.1186/s13018-024-04580-8.

## Introduction

Osteoarthritis (OA) is the most prevalent debilitating joint disease characterized with the articular cartilage degeneration, osteophyte formation, subchondral bone sclerosis, and synovial hyperplasia, influencing 10–20% of the population worldwide [1–4]. It causes considerable economic and societal burdens as its high morbidity and disabling symptoms, leading to a total healthcare cost presently evaluated to be € 400 billion annual in Europe [5, 6]. The present OA therapy depends on its severity. For low-to-mild stage OA, the utilization of painkillers, intra-articular injections of hyaluronic acid or corticosteroids, and nonsteroidal anti-inflammatory drugs, can relieve the pain, yet these treatments do not hinder disease progression. The gold standard treatment for end-stage OA is the total joint arthroplasty. However, it is invasive and expensive [7]. Currently, there is no curative strategy for OA treatment since the pathogenesis of OA is far from clear [8]. Therefore, it is imperative to investigate the underlying molecule mechanism of OA to find out effective therapeutic targets.

Heat-shock protein beta1 (HSPB1), also known as HSP27, is a member of the small HSP family, containing ubiquitously expressed molecular chaperones that keeps cellular proteostasis under stress situations [9]. As previously reported, the small HSP family are capable of binding to unfolded proteins preserving them in a folding-competent state, whose release requires cooperation with ATP-dependent HSPs [10]. In addition, HSPs also demonstrate the capacity to serve as RNA-binding proteins (RBPs). Davila et al. suggested that HSP27 alleviated oxidative stress-induced neuronal injury through binding to the 3′ UTR of bim mRNA [11]. HSP27 negatively mediated endogenous apoptosis through binding to cytochrome c [12]. Sinsimer et al. found that chaperone HSP27 as a subunit of ASTRC shows high-affinity AU-rich element (ARE) binding activity, and its knockdown contributed to proinflammatory cytokine ARE-mediated mRNA stabilize [13]. All of these studies identified HSPB1 as an RBP. Nevertheless, the profile of HSPB1 bound RNA is unknown.

Evidence demonstrated that HSP27 is downregulated in OA chondrocytes, and it shows a prominent function in the articular chondrocyte homeostasis through regulating cellular metabolism or interleukin (IL)-1beta-induced IL-6 secretion [14, 15]. However, its underly molecular regulation mechanism in OA needs further elucidate. In this work, we explored the profile of HSPB1 bound RNA and reveal the potential regulation mechanism of HSPB1 in OA using improved RNA immunoprecipitation and sequencing (iRIP-seq). iRIP-seq is a recently developed technology that can capture RBP-bound RNA targets directly and indirectly [16]. In the method of iRIP-seq, UV cross-linking of cellular protein-RNA interactions, RNase digestion of naked RNA regions, and immunoprecipitation of the protein-RNA complex were used to obtain the RNA–protein complexes including direct and indirect RNA–protein interactions, followed by steps of RNA isolation, cDNA library construction, and Illumina sequencing [17]. The results demonstrated that HSPB1 may be involved in the progression of OA through binding to PLEC, ROR2, EGFR, and COL5A1.

## Materials and methods

### Cell culture and plasmid overexpression

HeLa cells were incubated at 37 ℃ in Dulbecco’s modified eagle’s medium supplemented with 10% fetal bovine serum, streptomycin (100 g/mL), and penicillin (100 U/μl). The cells were transfected with a HSPB1-overexpressing plasmid using Lipofectamine 2000 (Invitrogen, Carlsbad, CA, USA).

### Co-immunoprecipitation

HeLa cells were first lysed in ice-cold lysis buffer (1 × PBS, 0.5% sodium deoxycholate, 0.1% SDS, 0.5% NP40) with RNase inhibitor (Takara, 2313) and a protease inhibitor (Solarbio, 329–98–6) on ice for 5 min. The mixture was then vibrated vigorously and centrifuged at 13,000 × g at 4℃ for 20 min to remove cell debris. The supernatant was incubated with DynaBeads protein A/G (Thermo, 26,162) conjugated with anti-flag antibody (Sigma, F1804) or normal IgG at 4 ℃ overnight. The beads were washed with low-salt wash buffer, high-salt wash buffer and 1X PNK buffer, respectively. Resuspend the beads in elution buffer and then divided into two groups, one for RNA isolation from HSPB1-RNA complexes and another for the western blotting assay for HSPB1.

### Western blot

Resuspend sample with 40 ul elution buffer (50 mM Tris–Cl (pH = 8.0), 10 mM EDTA (pH = 8.0), 1%SDS and incubate it at 70 °C for 20 min at 1,400 rpm. Centrifuge the sample at 13,200xg for a short time. Transfer the supernatant to a new EP tube, while the tube is on the magnetic separator. After that complexes were eluted by boiling for 10 min in boiling water with 1X SDS sample buffer and separated on 10% SDS-PAGE. With TBST buffer (20 mM tris-buffered saline and 0.1% Tween-20) containing 5% non-fat milk power for 1 h at room temperature, membranes were incubated with primary antibody: Flag antibody (1:2,000, Sigma, F7425), actin (1:2000, CUSABIO) and then with HRP-conjugated secondary antibody. Bound secondary antibody (anti-mouse or anti-rabbit 1:10,000) (Abcam) was detected using the enhanced chemiluminescence (ECL) reagent (Bio-Rad, 170,506).

### iRIP-seq library preparation and sequencing

The HSPB1-bound RNAs were isolated from the immunoprecipitation of anti-Flag using TRIzol (Invitrogen). Complementary DNA (cDNA) libraries were prepared with the KAPA RNA Hyper Prep Kit (KAPA, KK8541) based on the provided procedure and high-throughput sequencing of the cDNA libraries was performed on an Illumina Xten platform for 150 bp paired-end sequencing.

### Data analysis

After reads were aligned onto the genome with TopHat 2 [18], only uniquely mapped reads were used for the following analysis. “ABLIRC” strategy was used to identify the binding regions of HSPB1 in genome [19]. Reads with at least 1 bp overlap were clustered as peaks. For each gene, computational simulation was used to randomly generated reads with the same number and lengths as reads in peaks. The outputting reads were further mapped to the same genes to generate random max peak height from overlapping reads. The whole process was repeated for 500 times. All the observed peaks with heights higher than those of random max peaks (*p*-value < 0.05) were selected. The IP and input samples were analyzed by the simulation independently, and the IP peaks that have overlap with input peaks were removed. The target genes of IP were finally determined by the peaks, and the binding motifs of HSPB1 protein were called by HOMER software [20].

### Functional enrichment analysis

To sort out functional categories of peak associated genes (target genes), gene ontology (GO) terms and KEGG pathways were identified using KOBAS 2.0 server [21]. Hypergeometric test and Benjamini–Hochberg FDR controlling procedure were used to define the enrichment of each term.

### Tissue preparation

Individuals were eligible if they had pain or stiffness of the knee, limited joint movement, and joint atrophy of the muscles around the knee. Patients with the presence of a clear pathological condition rather than OA that could explain the existing complaints, such as other rheumatic disease, previous knee joint replacement, congenital dysplasia, osteochondritis dissecans, intra-articular fractures, septic arthritis, Perthes’ disease, ligament or meniscus damage, and Baker’s cyst, and comorbidity that did not allow physical evaluation were excluded [22, 23]. All the enrolled patients were aged 55–75 years with severe OA evaluated according to the Lequesne index. Knee tissues were collected from the patients with OA during surgical operation and maintained at -80 ℃ for subsequent RNA isolation and protein extraction. Knee tissues from patients without OA were classified as control group. This study was approved by the Ethics Committee of the Jiangxi Provincial People's Hospital, and all the enrolled patients were informed consent.

### RT-qPCR

Total RNA was extracted from knee tissues using trizol (Invitrogen, 15,596,018) and reverse transcribed into cDNA using RevertAid First Strand cDNA synthesis Kit (Invitrogen, K1622). qPCR was performed to detect the expression of HSPB1 and other genes using QuantityNova SYBR Green PCR Kit (QIAGEN, 208,054), with relative mRNA expression being evaluated by 2^−△△Ct^ methodology and β-actin serving as internal reference. The primer sequences are compiled in Additional file 1: Table S1.

### Immunoprecipitation

RIP determination was conducted using Magna RIP Kit (Merck Millipore, 17–701) based on corresponding instructions. The obtained knee tissues were dispersed into individual cells and lysed, and then incubated with magnetic conjugated anti-HSPB1 or control anti-immunoglobulin G (IgG) at 4 ℃ overnight. After purification, RT-qPCR was performed to determine EGFR, PLEC, COL5A1, and ROR2 relative fold enrichment. The primer sequences are compiled in Additional file 1: Table S1.

### Statistical analysis

Statistical analyses were conducted with SPSS software (Chicago, IL, USA; version 22), and outcomes are shown as mean ± standard deviation of at least three experiments. Significance between groups was detected using Student’s *t* test, and a *p*-value < 0.05 was considered as statistical significance.

## Results

### Qualified sequencing data were obtained for downstream analysis

**Fig. 1:**
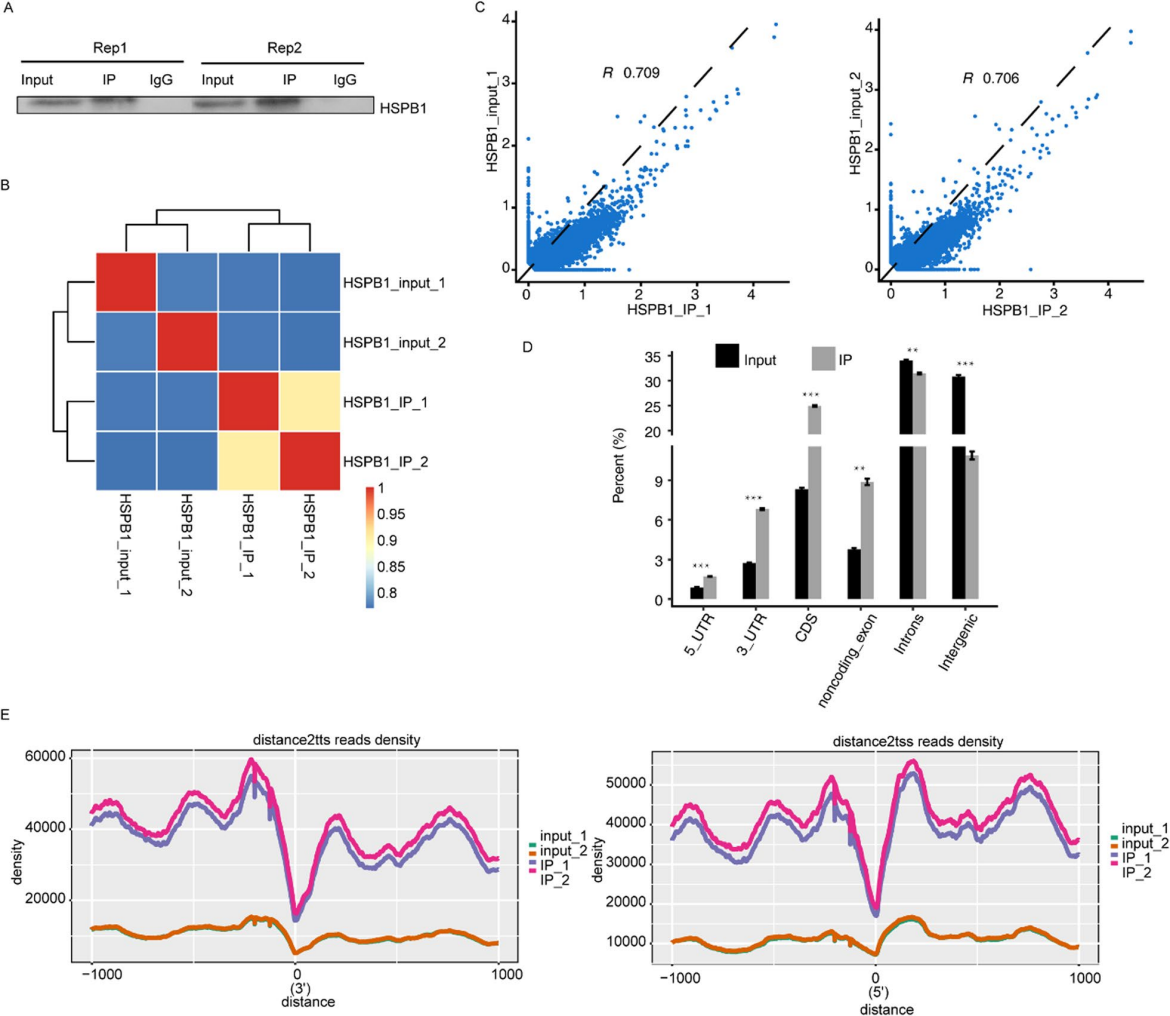
Qualified sequencing data were obtained for downstream analysis. **A** Western blot analysis of HSPB1 immunoprecipitates using anti-flag monoclonal antibody. Two replicates were performed. **B** Heatmap clustering analysis of sample correlation based on the normalized mapped reads on each gene, showing two immunoprecipitated samples clustered. **C** Scatter plot showing Pearson correlation between immunoprecipitated and input samples. **D** Percent of mapped reads on different genomic regions. **E** Peak reads density within 1 kilobase (kb) from the transcription start site (TSS) and from the transcription termination site (TTS)

**Fig. 2:**
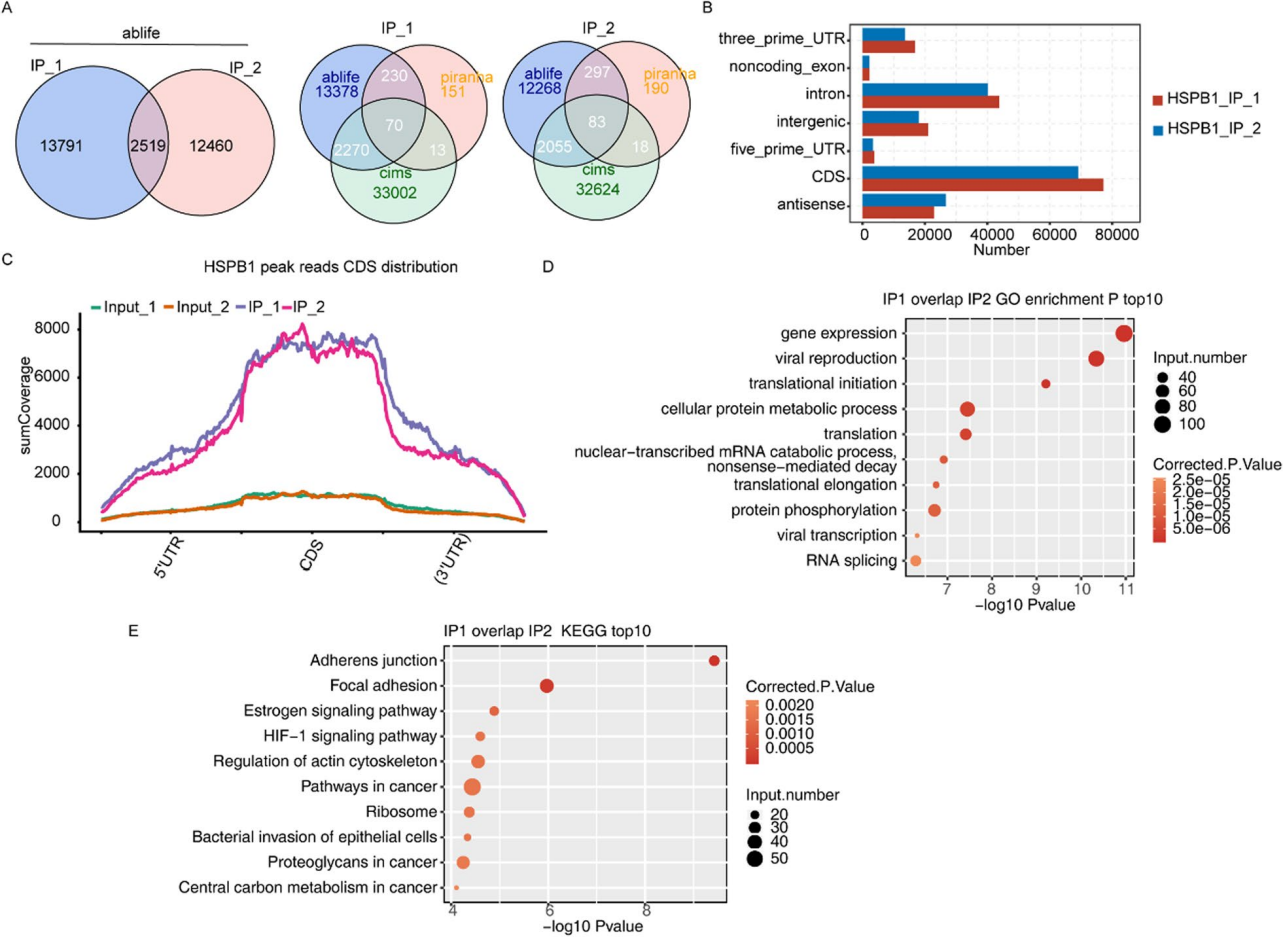
Analysis of the HSPB1-binding peaks. **A** Left Venn diagram showing the overlap of HSPB1 bound peaks obtained from two replicates of iRIP-seq which were called by ABLIRC algorithm. Right Venn diagram showing the overlap of HSPB1 bound peaks called by ABLIRC, cims and piranha methods, respectively. **B** Percent of predicted peaks on different genomic regions. **C** Peak reads density in 5′ UTR, CDS and 3′ UTR based on ABLIFE method. The reads density of HSPB1 peaks in all genes were plotted. **D** The top 10 enriched GO biological processes of the HSPB1-bound genes. **E** The top 10 enriched KEGG pathways of the HSPB1-bound genes

To investigate the HSPB1-RNA-binding activity and its potential contribution to OA, we obtained a transcriptome-wide interaction profile of HSPB1 in HeLa cells using iRIP-seq method. The flag-tagged HSPB1 was utilized for immunoprecipitation, and two independent iRIP replicates were carried out. Western blot detection demonstrated that Flag-HSPB1 protein was observed both in the total cell lysate and the IP specimens, but not in the IgG control (Fig. 1A). The prepared cDNA libraries from anti-flag IP and the total cell lysate (input) control were sequenced using the Illumina HiSeq X Ten system. A total of 24,557,586 and 34,854,589 clean reads for the replicate 1 of anti-Flag IP and input control were obtained, respectively, and 22,104,701 and 33,106,061 clean reads were obtained from those of replicate 2. The percentage of Q20 > 96%, Q3 > 91%, and GC ranged from 57 to 62%, indicating the high quality of the data. Thereafter, the reads were processed and aligned onto the human GRCH38 genome using Tophat2, approximately 69–87% of them were mapped and approximately 8–23% were uniquely mapped. A larger faction of uniquely mapped reads contained the spliced junction in IP samples than that in the input controls (Table 1). Heatmap clustering analysis of sample correlation based on the normalized mapped reads on each gene demonstrated that the two immunoprecipitated samples were clustered together and well-separated from the input samples (Fig. 2B). Scatter plot of the detected gene level in each pair of the samples showed that transcripts from an obvious fraction of genes were enriched in immunoprecipitated samples (Fig. 2C). Collectively, these results supported the success of the iRIP-seq experiments.

**Table 1:** Sample details from iRIP-seq and clean reads mapping on the reference genome

Sample ID	HSPB1_IP_1	HSPB1_IP_2	HSPB1_input_1	HSPB1_input_2
Raw	25,957,138	25,957,138	36,144,380	34,484,986
Clean	24,557,586	22,104,701	34,854,589	33,106,061
Clean percentage	94.61%	95.04%	96.43%	96.00%
Q20 percentage	96.72%	96.78%	97.01%	97.07%
Q30 percentage	91.60%	91.71%	92.27%	92.40%
GC percentage	62.00%	62.00%	57.00%	57.00%
Total mapped	21,456,099 (87.37%)	19,241,555 (87.05%)	24,309,491 (69.75%)	23,087,106 (69.74%)
Total uniquely mapped	1,761,223 (8.21%)	1,679,030 (8.73%)	5,660,856 (23.29%)	5,321,046 (23.05%)
Total multiple mapped	19,694,876 (91.79%)	17,562,525 (91.27%)	18,648,635 (76.71%)	17,766,060 (76.95%)
Rmdup reads	896,546 (50.9%)	854,603 (50.9%)	3,753,096 (66.3%)	3,597,729 (67.61%)

Furthermore, genomic location analysis of the aligned reads from the immunoprecipitated and control samples suggested that the HSPB1-related reads were notably enriched in the 5′ UTR, 3′ UTR, CDS, and noncoding exon, whereas depleted in the intergenic and introns regions compared to those of control samples (Fig. 1D). In addition, we analyzed the distribution of peak reads density within 1 kilobase from the transcription start site (TSS) and from the transcription termination site (TTS), indicating that peaks enrich in upstream of TTS (Fig. 1E).

### Analysis of the HSPB1-binding peaks

The rmdup reads that uniquely mapped to genomic locations and removed PCR duplicates were chosen for peak calling and further analysis. A total of 896,542 and 3,753,096 usable reads for the replicate 1 of anti-Flag IP and input control were obtained, respectively, and 854,603 and 3,597,729 reads were obtained from those of replicate 2 (Table 1). The peaks from HSPB1 iRIP-seq reads were analyzed using ABLIFE algorithm, and a total of 2519 HSPB1-binding peaks sharing overlapped genomic locations were obtained between the two replicates (Fig. 2A, left). In addition, Piranha and CIMS were also utilized to capture HSPB1-related peaks, demonstrating that the CIMS peaks had a better overlap with ABLIFE peaks (Fig. 2A, right). The majority of the obtained peaks were located on CDS (Fig. 2B). Further analysis demonstrated that the densities of the CDS peak reads were generally higher than those of the 5′ UTR and 3′ UTR peaks (Fig. 2C). Moreover, GO analysis of the obtained peaks from the two IP replicates revealed involvement in several terms including gene expression, translation initiation, cellular protein metabolic process, and nonsense-mediated decay (Fig. 2D), and KEGG analysis of those overlapped peaks demonstrated involvement in adherens junction and HIF-1 signaling pathway (Fig. 2E).

### Analysis of the HSPB1-binding motifs

**Fig. 3:**
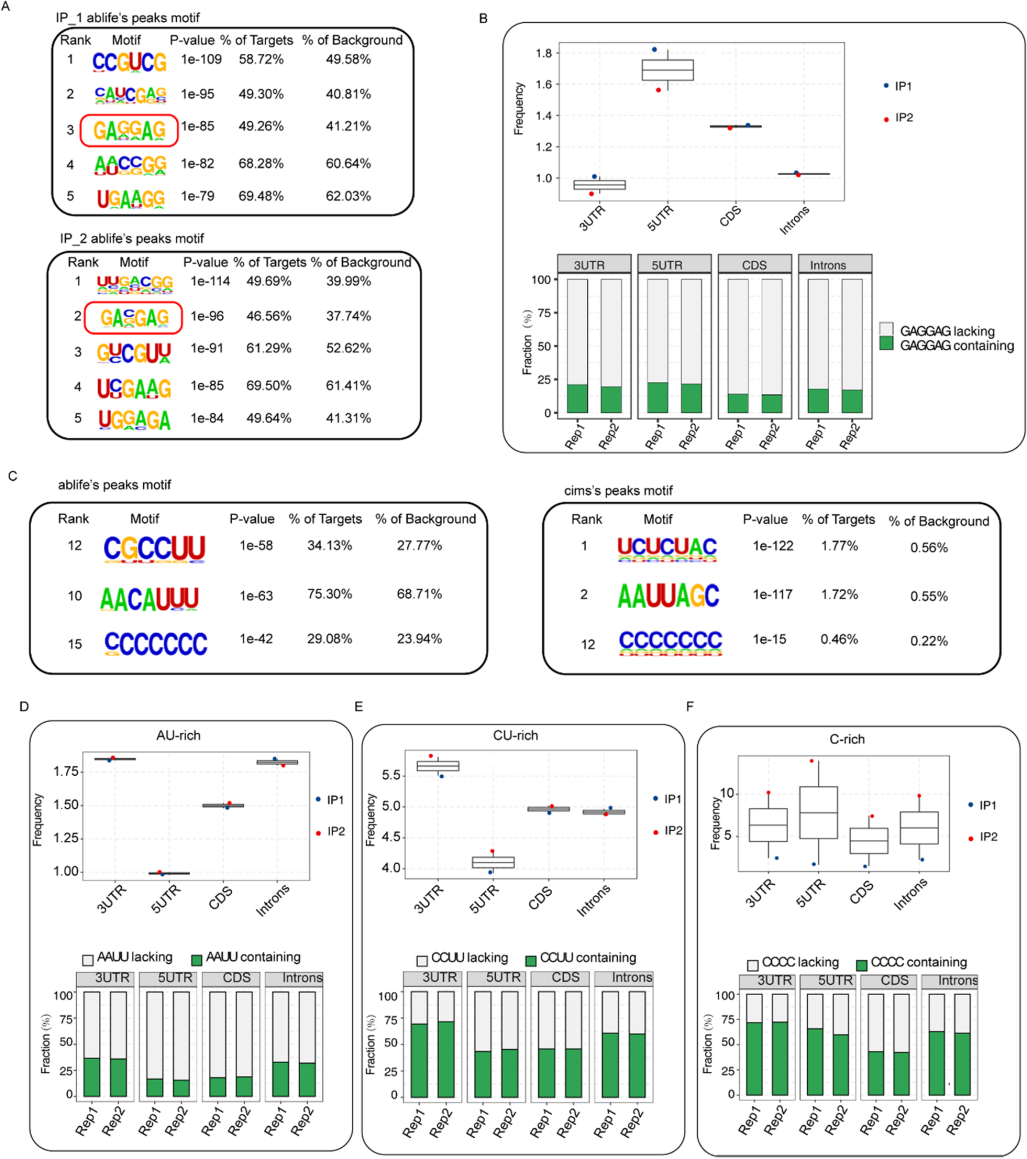
Analysis of the HSPB1-binding motifs. **A** Over-represented motifs in HSPB1 bound peaks from the two IP replicates. **B** The frequency of GAGGAG motif was higher in 3′ UTR and 5′ UTR regions in human HeLa cells. Box plot (up) showing the GAGGAG motif frequency in HSPB1-binding peaks which was located in different gene region including 3′ UTR, 5′ UTR, CDS and intronic. The frequency was calculated by hit motif length per kilo base of peaks. The barplot (down) showed the proportions of HSPB1 peaks with GAGGAG motifs in relative to those without. **C** AU-rich, CU-rich and C-rich motif were identified by ABLIRC and cims methods. **D** The frequency of AU-rich motif was higher in 3′ UTR and intronic regions in human HeLa cells. Box plot (up) showing the AU-rich motif frequency in HSPB1-binding peaks which was located in different gene region including 3′ UTR, 5′ UTR, CDS and intronic. The frequency was calculated by hit motif length per kilo base of peaks. The barplot (down) showed the proportions of HSPB1 peaks with AU-rich motifs in relative to those without. **E** The frequency of CU-rich motif was higher in 3′ UTR region in human HeLa cells. Box plot (up) showing the CU-rich motif frequency in HSPB1-binding peaks which was located in different gene region including 3′ UTR, 5′ UTR, CDS and intronic. The frequency was calculated by hit motif length per kilo base of peaks. The barplot (down) showed the proportions of HSPB1 peaks with CU-rich motifs in relative to those without. **F** The frequency of C-rich motif did not show significant differences on genome region in human HeLa cells

Furthermore, HOMER software was conducted to identify the sequence motifs enriched in HSPB1 bound peaks from the two IP replicates. Obviously, the sequence of GAGGAG was over-represented in the peaks identified from per independent IP replication (Fig. 3A). Further frequency analysis suggested that GAGGAG was present in different gene region including 3′ UTR, 5′ UTR, CDS and introns, and its frequency in the CDS and 5′ UTR was slightly higher than that in 3′ UTR and introns regions (Fig. 3B). As well, ABLIFE and CIMS algorithm indicated that HSPB1 bound to AU-rich motifs (Fig. 3C), which was consistent with previous study [13]. CU-rich and C-rich motif were also identified among the binding motifs (Fig. 3C), and the binding characteristics were similar to that of its interacting protein PCBP1 [9]. Frequency analysis suggested that all AU-rich, CU-rich, and C-rich were present in several gene regions including 3′ UTR, 5′ UTR, CDS and introns (Fig. 3D–F), and the proportion of peaks containing AU-rich, CU-rich, or C-rich motif in 3′ UTR was slightly higher than that in other regions (Fig. 3D–F).

### The reads density landscape of HSPB1-binding peaks on OA-related gene transcripts and additional confirmation

**Fig. 4:**
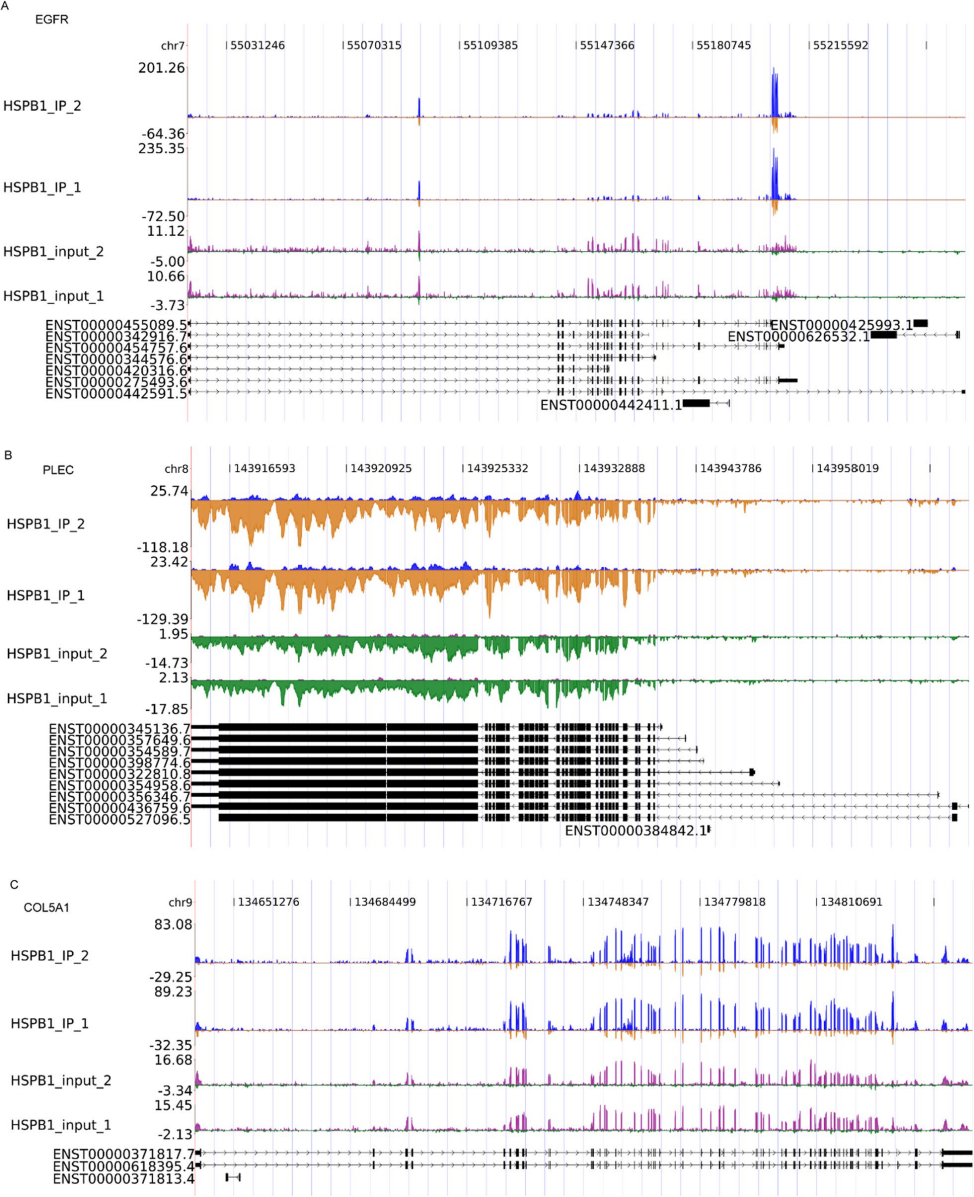
Reads density landscape of HSPB1-binding peaks on osteoarthritis-related gene transcripts. **A** EGFR. **B** PLEC. **C** COL5A1

**Fig. 5:**
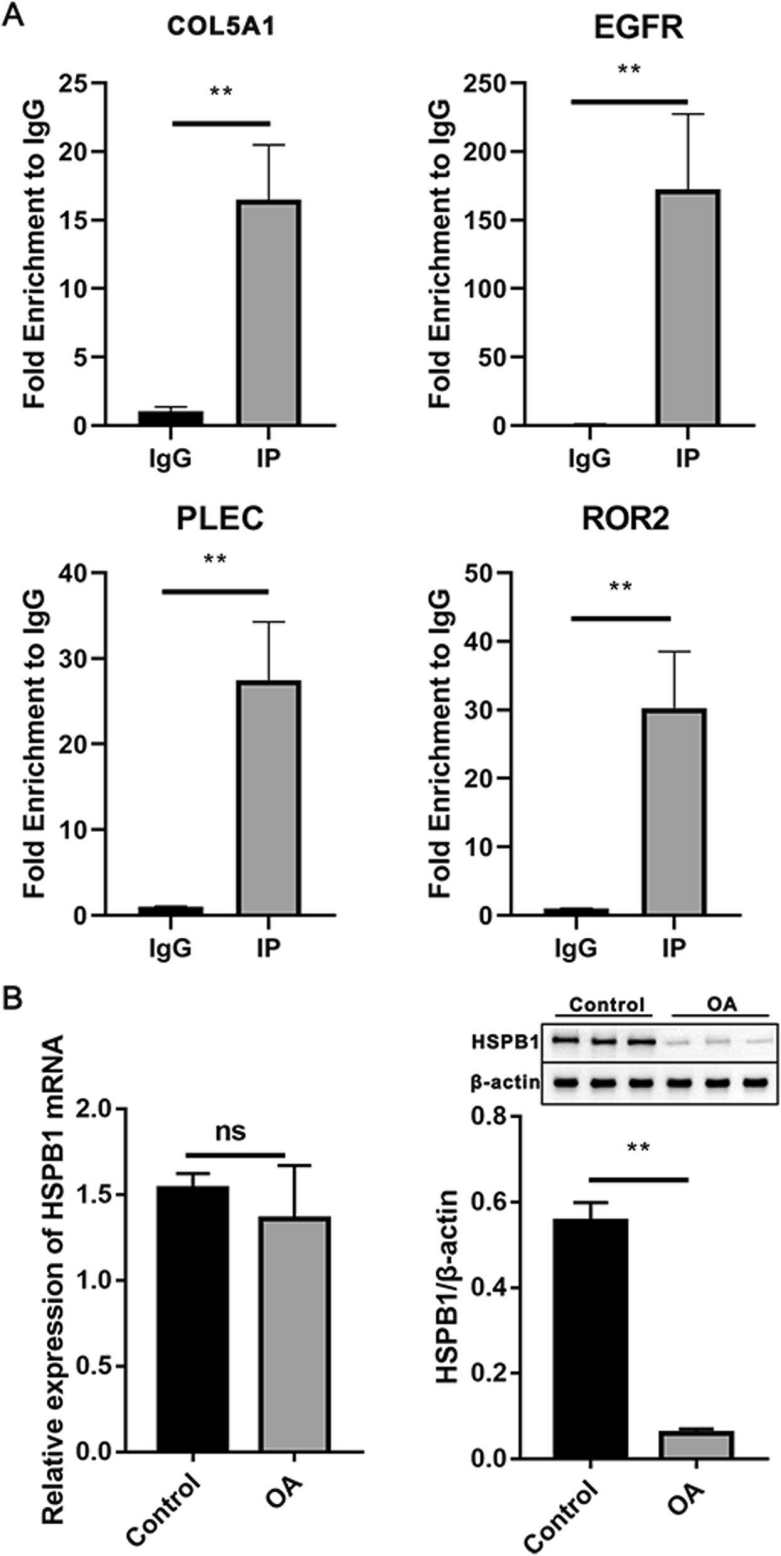
HSPB1 binds to OA-associated genes and is low expression in OA tissues. **A** RIP–qPCR validation of HSPB1-bound genes. **B** Relative expression of HSPB1 in OA tissues. Data are shown as mean ± SD of at least three repetitions. *p < 0.05, ** *p* < 0.01, ns, non-significant

Next, we analyzed the HSPB1-binding targets through corresponding peaks, finding several gens associated with OA including EGFR (Fig. 4A), PLEC (Fig. 4B), COL5A1 (Fig. 4C), and ROR2 (Additional file 1: Figure S1). The association of EGFR, PLEC, COL5A1, and ROR2 mRNA to HSPB1 was additionally confirmed in OA tissues by the quantitative RIP-PCR experiments (Fig. 5A). In addition, HSPB1 protein were fund to be downregulated in OA tissues compared to that in healthy control (Fig. 5B).

## Discussion

HSPB1, a subtype of the small HSP family, is involved in multiple biological processes containing macrophage infiltration [24], tumor proliferation and metastasis [25], mitochondrial dysfunction [26], epithelial-mesenchymal transition [27], as well as endoplasmic reticulum stress [28]. In addition, several studies have identified HSPB1 as an RBP associated with oxidative stress, apoptosis, and inflammatory [11–13]. However, the profile of HSPB1 bound RNA is far from clear. In this work, we captured an unbiased HSPB1-RNA interaction map in Hela cells using the iRIP-seq method. The results demonstrated that HSPB1 interacted with plentiful of mRNAs and genomic location toward the CDS region, suggesting its function in translational regulation of the bound mRNA. Moreover, functional enrichment of HSPB1-related peaks showed that overlapped peaks of two repetitions were involved in gene expression, translation initiation, and cellular protein metabolic process, consistent with previous study. As reported by Kuroyanagi et al., HSPB1 suppressed the translation initiation process of osteoblast‑like cells through interacting with eukaryotic translation initiation factor 4E, an mRNA cap‑binding protein that controls translation [29]. Wang et al. found that HSP27 contributes to high expression of HSPB8 in breast cancer cells, leading to proliferation and metastasis of cancer cells [30]. Additionally, HSP27 participated in cell cycle regulation of lung fibroblast cells through decreasing the expression of transcriptional repressors E2F-4 and p130 [31]. Vicente Miranda et al. revealed that HSP27 led to the generation of nontoxic α-synuclein species by decreasing methylglyoxal-induced cellular α-synuclein aggregation in vitro, thereby regulating the glycation-mediated cellular pathologies in synucleinopathies [32]. As well, the present study showed that HSPB1-related peaks were enriched in nonsense-mediated decay, a conserved regulatory mechanism for mRNA quantity and quality control in eukaryotic cells that implicates multiple human disease comprising neurodevelopmental disorders, cancer, and viral infections [33, 34]. It could be interesting to further investigate the regulatory effect of HSPB1 in nonsense-mediated decay.

Next, HOMER software analysis showed that HSPB1 bound peaks were over-represented in GAGGAG sequences, highly presented in the CDS and 5′ UTR regions. In addition, ABLIRC and CIMS algorithm indicated that HSPB1 bound to AU-rich motifs and the proportion of AU-rich peaks in 3′ UTR were slightly higher than that in other regions. 3′ UTR-localized AREs-bearing mRNAs encoding proteins required for intracellular signaling, cell proliferation, and inflammatory responses are maintained at low levels in monocytes dominantly due to their rapid degradation [35, 36]. Previous study has demonstrated HSPB1 as an ARE-binding protein vital for rapid degradation of ARE-bearing mRNA. As well, the study showed that HSPB1 bound to the TNF-α ARE and its inhibition contributed to dramatic stabilization of cytokine TNF-α [13]. Another study reported by Sommer et al. revealed that HSPB1 upregulation impeded the expression of ARE-mRNA encoding the c-Yes [37]. Our current study confirmed HSPB1 as an ARE-binding protein as previous studies, but its function as an ARE-binding protein requires further elucidate.

Moreover, we found several gens associated with OA among HSPB1-binding targets including EGFR, PLEC, COL5A1, and ROR2 and the association of these selected mRNAs to HSPB1 was additionally confirmed in OA tissues. In addition, we found the low expression of HSPB1 in OA tissues. Qin et al. demonstrated the remarkable reduction of EGFR at the early OA stage and EGFR deficiency accelerated Knee OA development in vivo [38]. EGFR activation persistently enlarged articular cartilage of mice due to the elevation of proliferation ability and survival rate of cartilage cells, whereas EGFR suppression impaired the protective function against OA in EGFR activation mice [39]. As reported by Rice et al., PLEC is a susceptibility gene of OA that encodes plectin, a cytoskeletal protein involved in Wnt signaling and immune modulation, which were found to have a function in OA [40, 41]. Bioinformatics analysis by Zhu et al. revealed the association of COL5A1 to OA, and experiment showed its differential expression between OA and matched control [42]. Thorup et al. reported that ROR2 blocking alleviated pain and promoted cartilage integrity in OA through inactivating yes-associated protein (YAP) signaling, indicating ROR2 as a promising therapeutic target for OA [43].

In conclusion, results from this unbiased HSPB1-RNA interaction profile analyzed using iRIP-seq demonstrated that HSPB1 interacts with plentiful of mRNAs toward the CDS region, suggesting its function in translational regulation of the bound mRNA. Moreover, we found several gens associated with OA among HSPB1-binding targets including EGFR, PLEC, COL5A1, and ROR2, and the association of these selected mRNAs to HSPB1 was additionally confirmed in OA tissues. However, this study is mainly based on bioinformatic analysis and lacks verification from cellular, animal, and clinical samples experiments. We will design additional studies in cellular, animal, and humans for a comprehensive understanding of the function of HSPB1 in OA. Overall, the current study provides a reliable insight to further investigate the molecular regulation mechanism of HSPB1 in OA.

### Supplementary Information

**Additional file 1: Fig. S1 **The reads density landscape of HSPB1-binding peaks on ROR2 transcripts. **Table S1** Primers information of RT-qPCR.

## Data Availability

Data supporting the findings of this study are available from the corresponding author upon reasonable request.
