# Supplementary material for: HSPB1 as an RNA-binding protein mediates the pathological process of osteoarthritis
Source: J Orthop Surg Res. 2024 Mar 1;19:156. doi: 10.1186/s13018-024-04580-8 (PMC10908047; doi:10.1186/s13018-024-04580-8)
Supplement: Supplementary file 1 — Additional file 1: Fig. S1 The reads density landscape of HSPB1-binding peaks on ROR2 transcripts. Table S1 Primers information of RT-qPCR. [file 13018_2024_4580_MOESM1_ESM.docx]

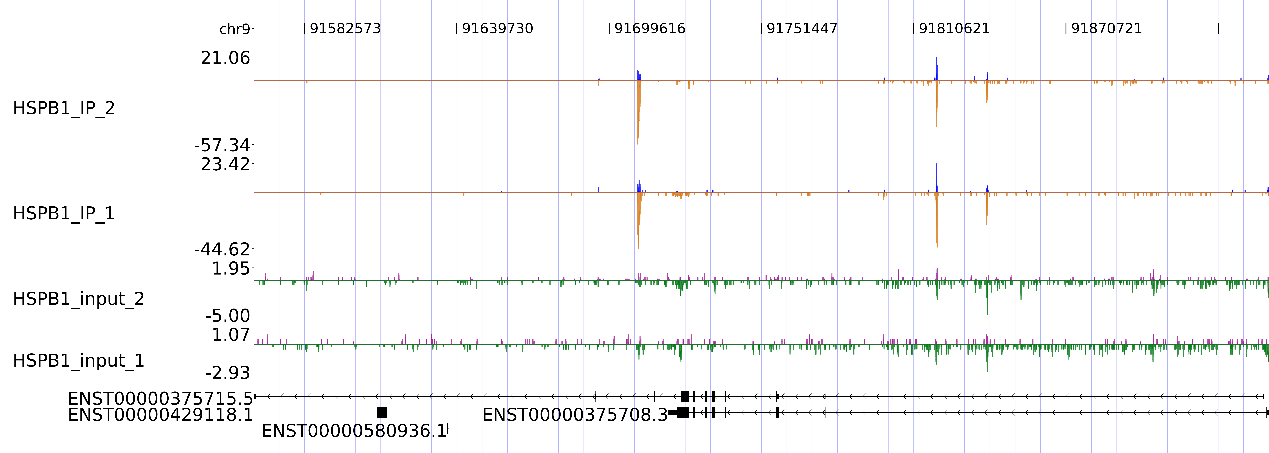


**Figure S1 The reads density landscape of HSPB1-binding peaks on ROR2 transcripts.**

Table S1 Primers information of RT-qPCR

| Primer name | Sequence (5’-3’) |
| --- | --- |
| HSPB1 Forward | ccaagtttcctcctccctgt |
| HSPB1 Reverse | gggatggtgatctcgttgga |
| EGFR Forward | cagcgctaccttgtcattca |
| EGFR Reverse | tgcactcagagagctcagga |
| COL5A1 Forward | gtggcacagaattgctctca |
| COL5A1 Reverse | aaacacgatgatgccattga |
| PLEC Forward | cgagaaggaacagctgaacg |
| PLEC Reverse | cagtcacctccacctgctta |
| ROR2 Forward | gggcaacctttccaactaca |
| ROR2 Reverse | cgttgctcacattgctcact |
| β-actin Forward | gcagaaggagatcactgccct |
| β-actin Reverse | gctgatccacatctgctggaa |
